# Lower limb joint biomechanics-based identification of gait transitions in between level walking and stair ambulation

**DOI:** 10.1371/journal.pone.0239148

**Published:** 2020-09-16

**Authors:** Martin Grimmer, Julian Zeiss, Florian Weigand, Guoping Zhao, Sascha Lamm, Martin Steil, Adrian Heller

**Affiliations:** 1 Institute for Sports Science, Technical University of Darmstadt, Darmstadt, Hesse, Germany; 2 Institute of Automatic Control and Mechatronics, Technical University of Darmstadt, Darmstadt, Hesse, Germany; 3 Technical University of Darmstadt, Darmstadt, Hesse, Germany; University of Pittsburgh, UNITED STATES

## Abstract

Lower limb exoskeletons and lower limb prostheses have the potential to reduce gait limitations during stair ambulation. To develop robotic assistance devices, the biomechanics of stair ambulation and the required transitions to level walking have to be understood. This study aimed to identify the timing of these transitions, to determine if transition phases exist and how long they last, and to investigate if there exists a joint-related order and timing for the start and end of the transitions. Therefore, this study analyzed the kinematics and kinetics of both transitions between level walking and stair ascent, and between level walking and stair descent (12 subjects, 25.4 yrs, 74.6 kg). We found that transitions primarily start within the stance phase and end within the swing phase. Transition phases exist for each limb, all joints (hip, knee, ankle), and types of transitions. They have a mean duration of half of one stride and they do not last longer than one stride. The duration of the transition phase for all joints of a single limb in aggregate is less than 35% of one stride in all but one case. The distal joints initialize stair ascent, while the proximal joints primarily initialize the stair descent transitions. In general, the distal joints complete the transitions first. We believe that energy- and balance-related processes are responsible for the joint-specific transition timing. Regarding the existence of a transition phase for all joints and transitions, we believe that lower limb exoskeleton or prosthetic control concepts should account for these transitions in order to improve the smoothness of the transition and to thus increase the user comfort, safety, and user experience. Our gait data and the identified transition timings can provide a reference for the design and the performance of stair ambulation- related control concepts.

## Introduction

Walking is one of the most fundamental movement tasks for humans. In addition to level walking, environments require adaptations of the lower limb to successfully navigate rough terrain or to ascend and descend slopes. An example of a human-made environment is the staircase. Based on two surveys [1, 2], it has been estimated that humans climb approximately 47 to 66 stairs per day [3]. While this is only a minor contribution to the 6000 to 7000 steps that humans perform per day [4], limitations in stair ambulation ability can limit accessibility at home, at the workplaces, or at public facilities. For those with limited or no walking capabilities [5], the environment can be adapted (e.g., elevators, ramps for wheel chair users). On the other hand, technologies such as prostheses [6, 7] or lower limb exoskeletons [8–11] can be used to regain the user’s ability of stair ambulation. Designing the hardware for these wearable lower limb robotics is likely to require the specification of the peak joint moments, the joint range of motion, and the average power [12]. Additionally, high-level robotic control requires information about the current and upcoming movement tasks. A change in the movement task-related control mode can be realized by switch-like mechanisms [13, 14], by scanning the environment [15, 16], or by predicting human intention based on real-time changes in lower limb movements [7]. Changing the robotic output kinematics and kinetics could also be accomplished by using reflex-like control based on human-like sensor feedback, and this would not require a change of the control mode [17].

While switches provide a high degree of safety, they do not allow for intuitive transitions. Scanning the environment could be a feasible solution for intuitive transitions if the sensors are not blocked by the user’s clothing (which can occur for lower limb prostheses). When using human movement for the detection of transitions, the transition timing must be identified. Previous work found that there is no characteristic biomechanical signature within two anticipatory level walking strides prior to ascent or descent of a staircase [18]. The identified changes in biomechanical variables were not different from those expected during the reduction of walking speed. As the anticipatory strides do not include characteristical biomechanical signatures, major kinetic and kinematic changes must occur within the first strides when entering a staircase.

This study aimed to capture human lower limb kinematics and kinetics of stair ambulation as a foundation for wearable robotic design and control. In particular, this article will focus on the transitions from level walking to stair ambulation as well as transitions from stair ambulation to level walking. Both transitions are investigated for stair ascent and descent at a medium stair slope. We target three specific questions with our analysis.

1. When comparing human kinematics and kinetics for level walking, stair ascent, and stair descent, previous studies revealed major differences [19–21]. Robots such as powered lower limb prostheses or exoskeletons therefore have to adapt their control to account for these changes. We want to know when these changes are initiated and completed to be able to design control algorithms that are able to mimic the transitions between level walking and stair ambulation. We believe that transition detection algorithms could use our results to evaluate if gait transitions can be successfully detected before or close to the required changes in joint biomechanics. As we analyze three joints, two limbs and four transitions, 24 transitions (beginnings and endings) exist. Our aim is not to formulate a specific hypothesis for each condition. However, we speculate that the limb that accomplishes the stride with the single stair height is more likely to begin the transition in the swing phase, as the center of mass movement is reduced, compared to the limb that accomplishes the first or last regular double stair height (which more likely starts in stance). If we assume that energy injection, dissipation, or redirection, as well as kinematic adaptation, are required, each limb’s transition could take place in some portion of a stance phase (energy changes) and some portion of a swing phase (kinematic adaptation). We therefore believe that transitions will end in the subsequent phase of either stance (if the transition began in swing) or swing (if the transition began in stance).

2. When changing the locomotion mode, transition phases could occur that do not match biomechanics of either level walking or stair ambulation. Thus far, it is unknown if such transition phases exist and how long they last. With this knowledge, wearable lower limb robotic developers could investigate solutions to mimic these transition phases for a seamless transition in between level walking and stair ambulation. While short transitions could avoid additional transition modes, long transitions could allow for reduced accuracy in detection timing, as biomechanical changes may not be that abrupt. Thus, long transition times might require less actuator power compared to sudden mode changes. We therefore would like to determine if transition phases exist and how long they last. We believe that transition phases exist for all lower limb joints. As hypothesized for the transition timing, transitions are expected either to begin in swing and end in the following stance, or to begin in stance and end in the following swing phase. Thus, the transitions at each limb are expected to last at least half of a gait cycle. As we do not consider walking velocity changes as a part of the mode transition (as it could simply be breaking), we do not believe that multiple strides at each limb are required to complete the transitions.

3. The detection of transitions between level walking and stair ambulation via kinetic or kinematic measures is possible after the initiation of the transition. This results in difficulties in transition detection, especially for devices such as powered lower limb prostheses that can only react on the movement of the residual part of the limb. In the best case, the initiation of a movement would be possible by volitional control. As such interfaces are still rare, an alternative could be to use kinematic measures of the contralateral limb or a proximal biological joint that initiates the transition first. Such information could be used to trigger the robotic joints without direct control of the user. We would like to investigate if there exists a consistent order for the transition of the hip, the knee, and the ankle joint, and if an order exists, how much time delay is present between these joints. We hypothesize that there is not a single order that can be found in all transitions as the required functionality during the transitions is too different. Due to the direct interaction and the coupling (biarticular structures) in between the limbs and the joints, we expect the relative time differences between the joints of interest to be less than half of a gait cycle for both the beginning and for the end of transitions.

## Materials and methods

### Subject information

This study recorded and analyzed barefoot walking kinematics and kinetics of 12 male subjects (25.4±4.5 yrs, 180.1±4.6 cm, 74.6±7.9 kg) who were, according to their oral feedback, free of gait-related impairments. The study protocol was approved by the institutional review board of the TU Darmstadt. All subjects provided written informed consent in accordance with the Declaration of Helsinki.

### Experimental setup

**Fig 1:**
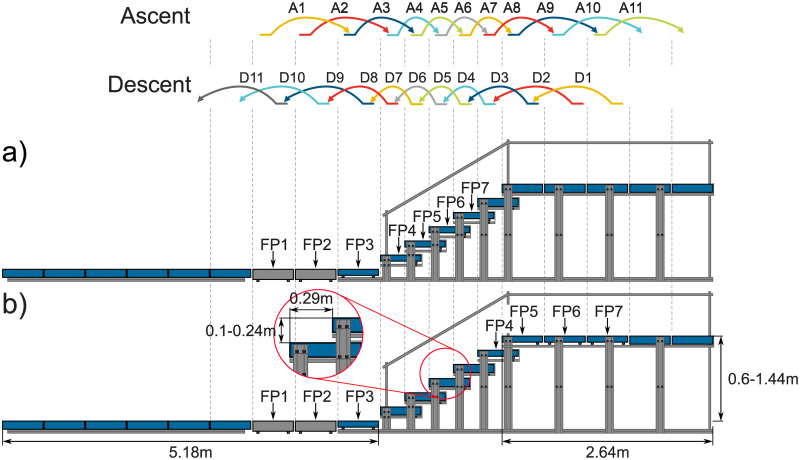
Stair and force plate configuration. Two different configurations of force plates (FP) were used to investigate on the four types of transitions in between level walking and stair ambulation. Setup a) covers most of the transition on the lower end of the stairs and setup b) covers most of the transition at the upper end. Both setups were used for the stair ascent (strides A1 to A11) and stair descent (strides D1 to D11). Due to size, FP1 and FP2 can not be placed on the staircase.

A 9.3 m long and 0.6 m wide walking track with an embedded staircase was used for the experiment. Seven force plates (five 9260AA and two 9287C, Kistler, Switzerland) were used in two configurations to measure ground reaction forces (GRF) at 1000 Hz (Fig 1). The force plate positions at the ground level (5.18 m in length) and the top level (2.64 m in length) were arranged individually so that we could measure the GRFs of each leg when approaching the staircase. A 3D motion capture system (18 Oqus, Qualisys, Sweden) recorded kinematics of 28 reflective markers at 180 Hz (see Appendix for marker positions), and all measurement systems were synchronized. Stair ambulation was performed at stair slopes of 19.0°, 30.4°, and 39.6°. While the stair depth was fixed to 0.29 m, stair height varied from 0.1 m (low), to 0.17 m (normal), and to 0.24 m (high), respectively. Only data from the normal height was used for the analysis in this article.

### Experimental protocol

Prior to the experiment, subjects walked up and down the staircase to familiarize themselves with the setup, to ensure each foot placement fell squarely within the force plates, and to determine their ideal starting locations. Subjects were instructed to walk as natural as possible with their preferred walking velocity and without targeting the force plates. If required, plate distances for the plates before and after the staircase were adapted to the subjects preferred step length. Following, the motion capture system was calibrated based on the manufacturer instructions for the 3D area of interest with the origin in the middle of the setup (FP6 in setup a). Subjects always began walking from a standing position and entered the staircase with the same foot (left foot for ascent and right foot for descent). Ten trials for each of both force plate setups (Fig 1) were performed at each stair height. Thus, in total 60 trials were performed by each subject where a single trial included both stair ascent and descent. Before each trial, the FP software removed the voltage offset for each plate (to ensure zero force without load) to avoid drifting force signals over the series of trials. Stair height was changed from either high to low or low to high, depending on the last setup of the previous subjects. Data recording began with approximately 3 s of quiet standing, at which point subjects were instructed to ascend the staircase. At the end of the top level, subjects stopped, turned around, and waited for a follow up signal (after 18 s total) to continue the trial and to descend the staircase. Upon reaching the end of the bottom level, the subjects stopped and waited until the trial was finished (35 s). For stair ascent, subjects entered the staircase at their seventh stride and performed four additional strides upon leaving the staircase (starting with the first stride at the top level). For stair descent, subjects entered the staircase at their fourth stride and performed seven additional strides upon leaving the staircase (starting with the first stride at the bottom level).

### Data processing

The motion capture data was labeled and tracked in the Qualisys Track Manager (see Appendix for marker placement). The kinematics and the kinetics (from BioWare, Kistler, CHE) were then exported for further processing in Matlab (MathWorks, US) and OpenSim. Joint kinematics (angles, angular velocities), kinetics (moment, power), and the center of mass (CoM) height and velocity were computed with OpenSim (version 3.3, [22]) using a full body model adapted from [23]. The hip, knee, and ankle angles in the saggital plane were low pass filtered with a conservative cutoff frequency of 20 Hz (2^nd^ order zero-lag Butterworth filter). The joint angular velocities were additionally filtered at 10 Hz. The joint moments were filtered at 10 Hz [24, 25]. The joint kinetics were normalized to the individual subject body mass. The joint moment rates (used in our statistical approach) of the hip, the knee, and the ankle were determined by numerical differentiation. The processed data was segmented based on the individual beginning (heel strike) and the termination (following heel strike same limb) of the stride. Following, the segmented data was normalized to one stride (gait cycle). For most of the level walking and stair ambulation strides, the beginning and the end of the stride was determined based on the vertical ground reaction force using a threshold of 20 N for the heel strike (toe strike) detection and 10 N for the toe off detection. After observing the data, the threshold levels were manually selected to avoid early or late detection due to force signal noise.

For stair ascent and descent, labels were allocated for stride identification. The foot that first contacted a force plate along the track (FP1) has the label A1 for stair ascent and the label D1 (corresponding to FP7 from setup b) for stair descent. With each additional step, label numbers increase until A11 (corresponding to FP7 from setup b) and D11 (corresponding to FP1) with the ground contact at the last force plate on the track (Fig 1). The end of the stride could not be determined by vertical GRF in the same setup (a, b, Fig 1) for some of the used strides. For the strides A10, D10, D11, and A11, the end of the stride was therefore determined based on the shank angular velocity zero-crossing, including the removal of the time offset (3% of the gait cycle) based on the findings of [26, 27]. As this approach did not work well for some of the subjects for the stair ambulation strides, another approach was required to determine the end of the stride for D3, D4, A6, and A7. We decided to use the second force plate setup to determine the end, as the required stair was instrumented in this setup. We used the extracted ground contact (heel or toe strike) timing based on the vertical GRF and determined the subject specific mean 3D velocity of both ankle markers at the same timing. This 3D velocity was previously filtered with a 4^th^ order zero-lag low pass Butterworth filter, with a cutoff frequency of 10 Hz. Individual mean 3D velocity thresholds were then used to identify the subject specific end of strides D3, D4, A6, and A7 based on the ankle marker kinematics that were processed in the same manner.

Subject means for all kinematic, kinetic, and spatio-temporal values were determined based on the ten trials per stride, and grand means were determined based on subject means. The duty factor describes the ratio of the stance phase and the swing phase as a percentage and is determined by the stance duration divided by the stride duration and multiplied by 100.

### Transition identification

A method was required to separate level walking from stair ambulation and to detect the beginning and the end of transition phases that do not match the kinematics and kinetics of these gaits. To have a repeatable approach without manually tuned variables and thresholds, we decided for an identification method based on statistics. A doubly repeated measures MANOVA (multivariate analysis of variance, [28]) was performed to identify the beginning and the end of transitions from level walking to stair ambulation and from stair ambulation to level walking (for both stair ascent and stair descent). The idea behind the MANOVA is to compare sample means of multiple dependent variables including the joint angle, the joint angular velocity, the joint moment, and the joint moment rate for the hip, knee, and ankle joint individually (*α* level of 0.05). The dependent variables of moment rate and joint angular velocity were specified as the time-normalized angle and moment data (to one stride) do not include time information nor the rate of change. While intersecting values of a joint angle could be similar, the joint angular velocity, and thus the behaviour, could be completely different. The statistical analyses was performed for each gait percent (one stride is normalized to 100 data points) of all eleven strides individually (A1 to A11 for ascent and D1 to D11 for descent). Strides A5 and A6 and strides D5 and D6 were used as the stair reference for ascent and descent, respectively, in order to test for a significant difference between ascent and descent strides. The states stair ascent or stair descent were considered if no difference was found between the analyzed gait percent (e.g., 34% of A4) and the same gait percent for one of the reference strides (A5 or A6 in this example).

With our setup, subjects were not able to reach their preferred walking velocity at the top level of the staircase. To account for the difference in the walking velocity, we used strides A1, D1, and D11 as our level walking reference. If the analyzed gait percent of a specific stride (e.g., 73% of D10) was not significantly different from at least one of these strides, this stride was considered to be level walking. If all reference strides (’either on stairs or during level walking) were significantly different, the related gait percent was considered to be a transition phase. To remove very short periods that were identified to be different from either level walking or stair ambulation, a moving average with a sliding window of 20% of the gait cycle (20 data points) was used to filter these results (for the complete ascending or descending portions of a trial). Such short periods often occurred near to heel strike, which was believed to be due to the different methods in partitioning the strides. The filtered state (ascent, descent, level walking, or transition) data improves the visualization and interpretation. The transition durations were determined as the duration from the start to the end of the transition (in gait percent, including stride transitions if occurred).

## Results

**Fig 2:**
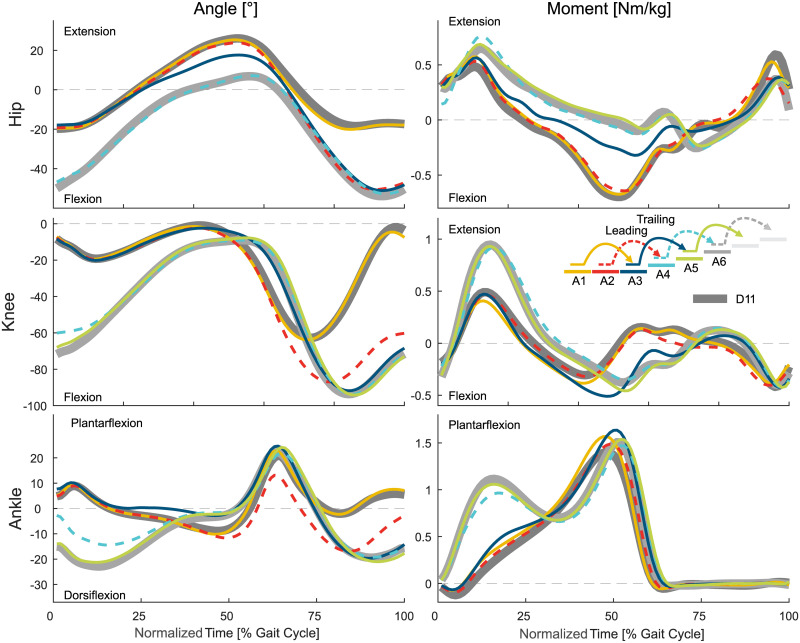
Biomechanics of level walking to stair ascent. Mean values of the hip, knee, and ankle angle and moment. For reference, the level walking stride D11 (thick, solid, dark gray) and the stair ascent stride A6 (thick, solid, light gray) are shown. Solid lines represent the left leg, dashed lines the right leg.

**Fig 3:**
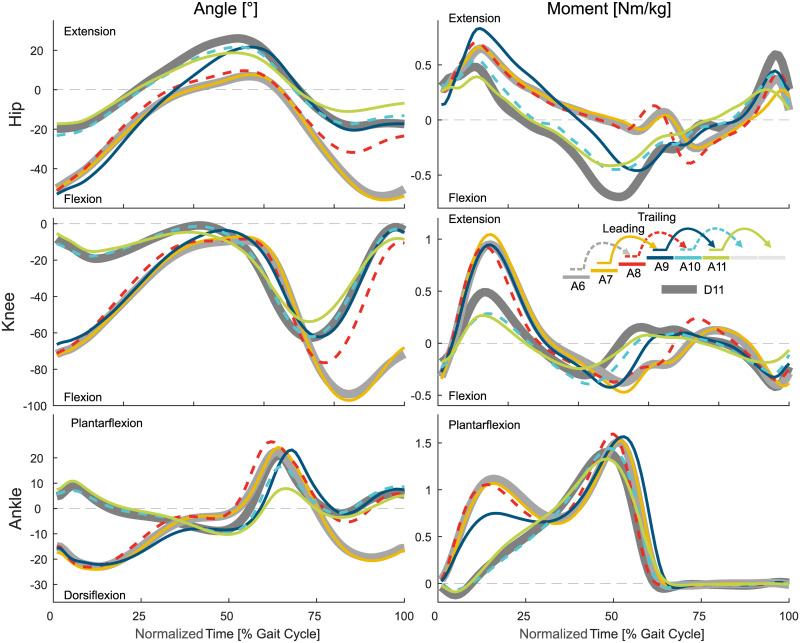
Biomechanics of stair ascent to level walking. Mean values of the hip, knee, and ankle angle and moment. For reference, the level walking stride D11 (thick, solid, dark gray) and the stair ascent stride A6 (thick, solid, light gray) are shown. Solid lines represent the left leg, dashed lines the right leg.

**Fig 4:**
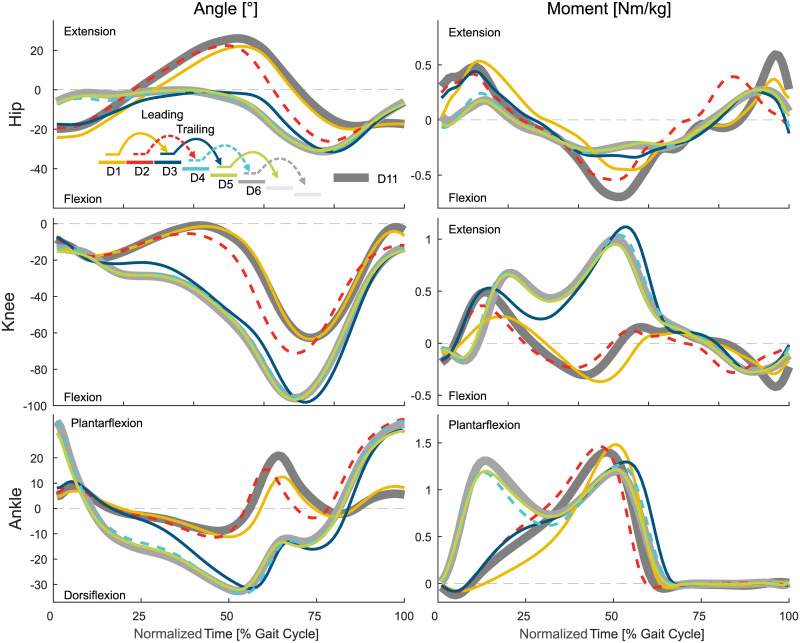
Biomechanics of level walking to stair descent. Mean values of the hip, knee, and ankle angle and moment. For reference, the level walking stride D11 (thick, solid, dark gray) and the stair ascent stride D6 (thick, solid, light gray) are shown. Solid lines represent the left leg, dashed lines the right leg.

**Fig 5:**
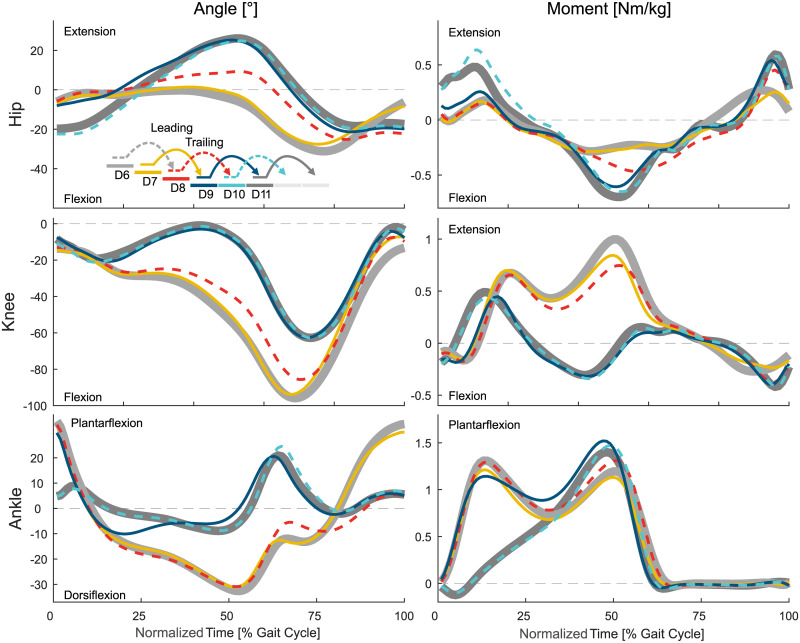
Biomechanics of stair descent to level walking. Mean values of the hip, knee, and ankle angle and moment. For reference, the level walking stride D11 (thick, solid, dark gray) and the stair ascent stride D6 (thick, solid, light gray) are shown. Solid lines represent the left leg, dashed lines the right leg.

**Fig 6:**
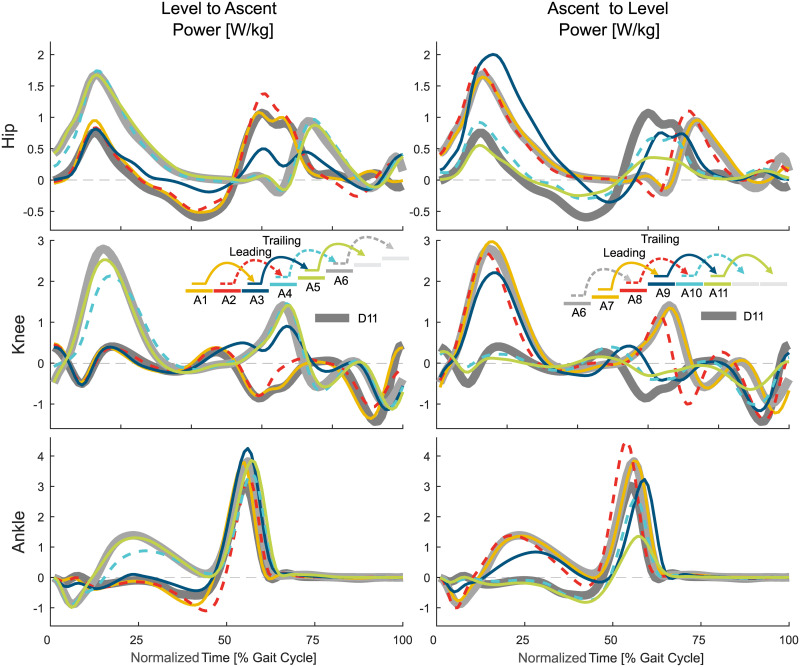
Joint power in the stair ascent transitions. Mean values of the hip, knee, and ankle power for the transition from level walking to stair ascent (left) and from stair ascent to level walking (right). For reference, the level walking stride D11 (thick, solid, dark gray) and the stair ascent stride A6 (thick, solid, light gray) are shown. Solid lines represent the left leg, dashed lines the right leg.

**Fig 7:**
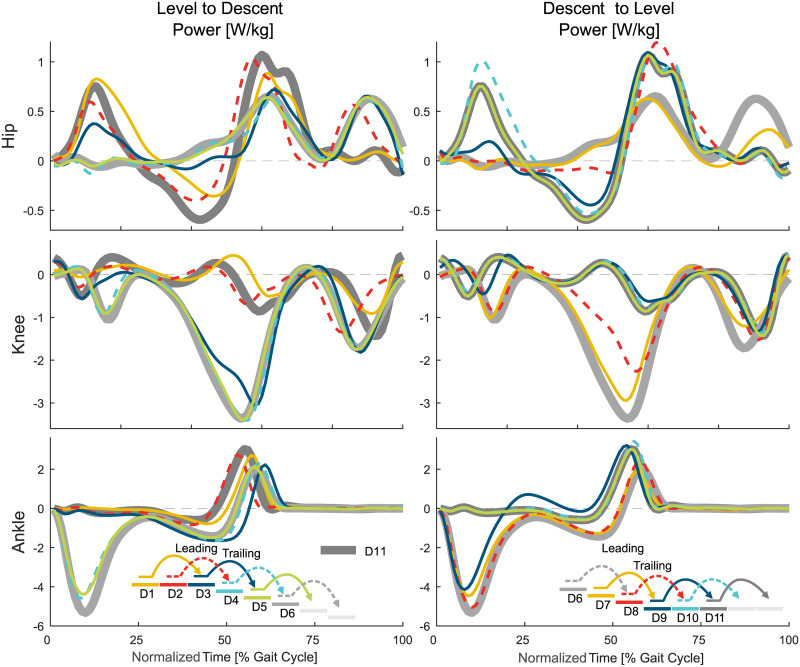
Joint power in the stair descent transitions. Mean values of the hip, knee, and ankle power for the transition from level walking to stair ascent (left) and from stair ascent to level walking (right). For reference, the level walking stride D11 (thick, solid, dark gray) and the stair ascent stride A6 (thick, solid, light gray) are shown. Solid lines represent the left leg, dashed lines the right leg.

**Fig 8:**
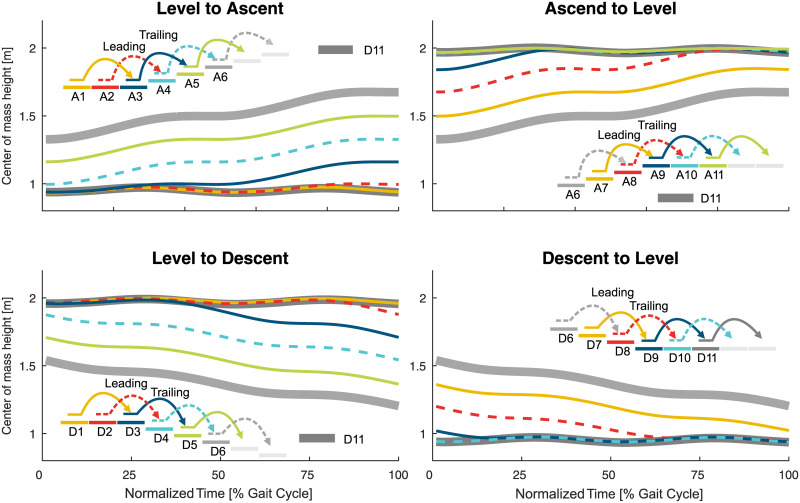
Center of mass height. Center of mass height for the level to ascend, ascend to level, level to descent, and descent to level transition. For reference, the level walking stride D11 (thick, solid, dark gray) and the stair ascent stride A6 (thick, solid, light gray) are shown. Solid lines represent the left leg, dashed lines the right leg. For visualization reasons, the center of mass height of D11 of the ascent to level and the level to descent transition was shifted to the height of the top level.

**Fig 9:**
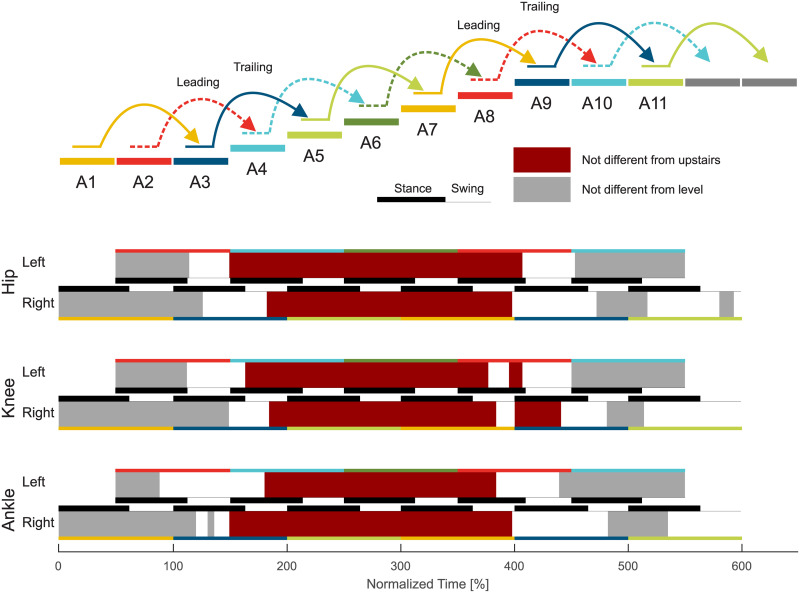
Statistical analysis of the ascent transitions. Joint specific (hip, knee, ankle) transitions from level walking to stair ascent and back to level walking for the strides A1 to A11. Individual stride phases that are not different from level walking (A1, D1, and D11) are indicated in gray. Phases that are not different from stair ascent (A5 and A6) are indicated in dark red. White areas are significantly different from level walking and from stair ascent.

**Fig 10:**
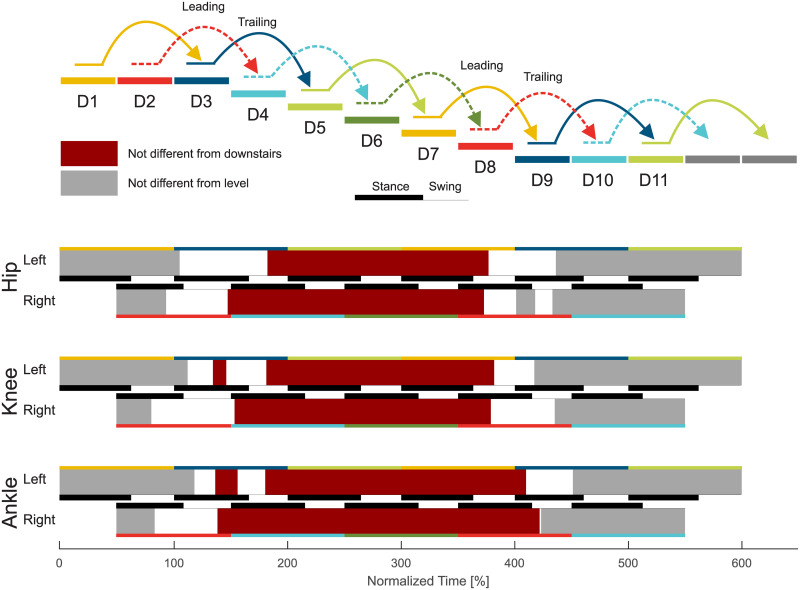
Statistical analysis of the descent transitions. Joint specific (hip, knee, ankle) transitions from level walking to stair descent and back to level walking for the strides D1 to D11. Individual stride phases that are not different from level walking (A1, D1, or D11) are indicated in gray. Phases that are not different from stair descent (D5 and D6) are indicated in dark red. White areas are significantly different from the level walking and from stair descent.

**Fig 11:**
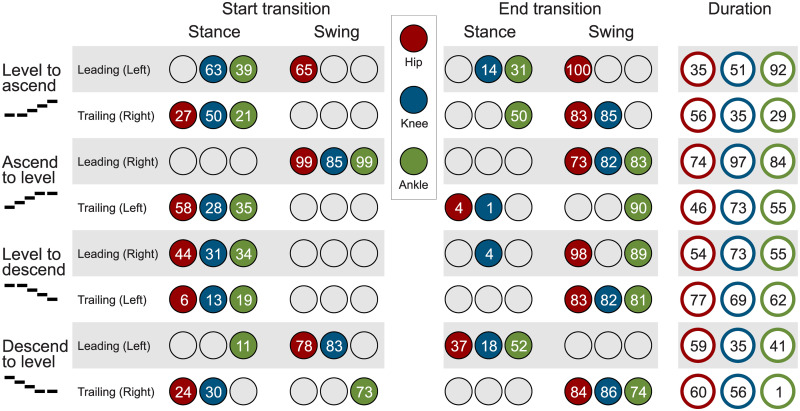
Joint specific timing. Joint specific timing of the transition start, the transition end, and the transition duration of the leading and the trailing limb for all analyzed types of transitions. Circle colors indicate the lower limb joint (hip in red, knee in blue, ankle in green). The numbers in the circles indicate the gait percent (in percent of the stride time) of the start and the end of the transition, and the total duration from the start to the end (sum of the passed gait percent in percent of the stride time).

**Fig 12:**
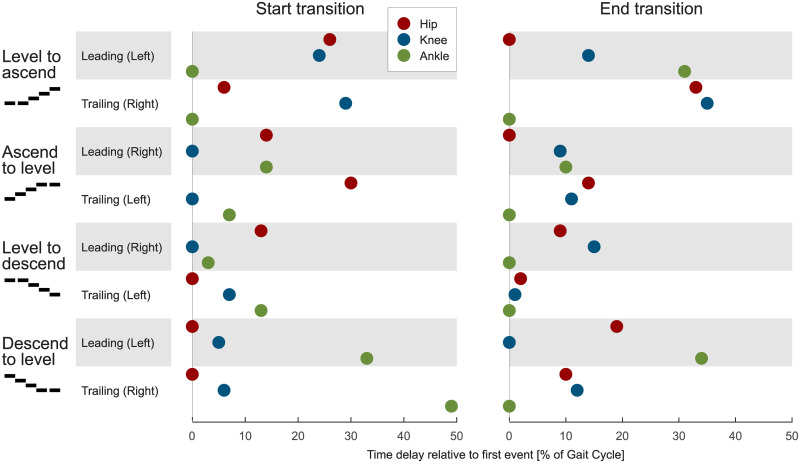
Relative time delay. Relative time delay in between the hip (red), the knee (blue), and the ankle (green) for the leading and the trailing limb of all transitions. The analyses was performed for the start (left) and the end (right) of the transition. The start occurs when leaving a state (e.g., level walking), and the end occurs when entering a new state (e.g., ascending stairs). The joint with the earliest transition is set to zero, and the other joints are placed relative to it based on their delay.

This section will describe the entry and exit transitions for both stair ascent and descent in terms of joint angles, joint moments, joint power, and CoM height (Figs 2 to 8). This data is supported by Figs 9 and 10 that present the statistical assessment comparing all strides (A1 to A11 and D1 to D11) to level walking (A1, D1, D11), stair ascent (A5 and A6), and stair descent (D5 and D6). Fig 11 presents the transition timing for stance and swing, and the duration of each transition, and Fig 12 highlights the transition timing in between the joints. Table 1 shows spatio-temporal characteristics of the transition strides.

**Table 1:** Grand means of the horizontal CoM velocity in forward walking direction, stride duration, stance duration and duty factor for the strides of the stair ascent (A) and the stair descent (D) trials.

Ascent											
Stride	A1	A2	A3	A4	A5	A6	A7	A8	A9	A10	A11
Velocity [m/s]	1.25	1.14	0.90	0.65	0.53	0.51	0.53	0.66	0.86	0.96	0.77
Stride duration [s]	1.12	1.11	1.11	1.12	1.12	1.16	1.18	1.29	1.26	1.19	1.24
Stance duration [s]	0.69	0.69	0.70	0.72	0.72	0.72	0.75	0.77	0.82	0.74	0.78
Duty factor [%]	61.2	62.4	63.2	63.7	64.1	62.5	63.2	59.8	64.8	62.1	63.4
Descent											
Stride	D1	D2	D3	D4	D5	D6	D7	D8	D9	D10	D11
Velocity [m/s]	1.01	0.95	0.75	0.59	0.56	0.56	0.64	0.83	1.08	1.21	1.24
Stride duration [s]	1.22	1.26	1.22	1.10	1.06	1.06	1.10	1.11	1.12	1.12	1.11
Stance duration [s]	0.76	0.73	0.81	0.72	0.68	0.69	0.69	0.72	0.68	0.70	0.68
Duty factor [%]	62.1	58.3	65.8	65.3	64.5	65.2	63.4	65.1	60.6	62.6	61.9

### Level to stairs ascent transition

#### Leading limb

The transition from level walking to stair ascent (Fig 2) starts with the leading limb (limb that enters staircase first, left) with a slight increase in ankle dorsiflexion in late midstance (A2, Fig 9). Major adaptations for the hip, knee, and ankle are seen at the beginning of the following swing phase. While the hip and knee transition to increased flexion as found in stair ascent, the ankle shows reduced plantarflexion that does not exist in level walking or stair ascent.

The transition for the leading limb is completed before midstance of the following stride (A4). The ankle completes the transition of the leading limb last. This is a result of the leading limb having only to ascent a single stair, which is accomplished with less dorsiflexion and a reduced plantarflexion moment compared to the continuous stair ascent gait.

#### Trailing limb

The transition of the trailing limb (limb that enters staircase second, right) starts during midstance (A3). The hip and ankle begin the transition earlier than the knee. The ankle shows reduced dorsiflexion and the hip has reduced extension. These changes are a result of the leading limb being one stair higher, which makes a level walking-like rollover impossible.

Next, the trailing limb ankle initiates the change of the CoM (Fig 8) direction within double support by increasing the push off (Fig 6). While the ankle finishes the adaptations within the stance phase of A3, the hip and knee continue to adapt until the middle of the swing phase.

### Stair ascent to level transition

#### Leading limb

The transition from stair ascent to level walking (Fig 3) starts at a similar timing for the leading limb (limb that leaves staircase first, right, A7) and the trailing limb (left, A8). From the middle of the swing phase of A7, statistical differences (Fig 9) were identified for the knee that are only slightly visible in the knee angle and joint moment. The transitions for the hip and the ankle follow with major adaptations in the kinetics (increased hip extension moment, reduced ankle plantarflexion moment) at the beginning of the consecutive leading limb stride (A9). Within A9, the hip extension may be increased to increase the horizontal walking velocity and to erect the trunk. As the trailing limb has still to be lifted to the top level, the stance phase of A9 remains more similar to that of stair ascent. Interestingly, while the knee showed the first slight adaptations during this transition to level walking, the primary adaptation occurs during late stance of A9. Late adaptations in A9 were also found for the ankle, which uses late plantarflexion (Fig 6) to accelerate the body on the top plateau of the staircase. The transition of the leading limb is complete during the swing of A9, which is earliest for the hip and is followed by the knee and the ankle. Additionally, Stride A11 was found to be different from the level walking strides starting in the early stance.

#### Trailing limb

The transition of the trailing limb begins during midstance of A8 (Fig 9) with small adaptations in the kinematics and kinetics for the knee and the ankle (Fig 3). While the stance phase of the trailing limb (A8) has an increased push off, the major transition occurs after toe off for all joints. The swing of A8 is used to reduce the joint flexion/dorsiflexion that was previously required during stair ascent. While the joint moment of the hip, knee, and ankle are comparable in this phase, the transition is obvious from the joint angles.

The trailing limb transition to level walking is first completed for the ankle during the late swing phase of A8. However, major kinematic changes remain present for the hip and the knee until the trailing limb heel strike during A10.

### Level to stairs descent transition

#### Leading limb

The transition from level walking to stair descent (Fig 4) begins with the leading limb (limb that enters staircase first, right, D2) during midstance (Fig 10). The transition of the ankle is delayed compared to the hip and/or the knee joint. The knee of the leading limb is first flexed, which is followed by the hip, with less extension to reduce stride length, and the ankle, with large plantarflexion to prepare for toe ground contact. The hip and the ankle primarily change their kinematics within the swing phase. The ankle push off (stance) is reduced, and the hip extension moments for braking the forward swing and for preparing for the heel strike increase (swing).

The transition of the leading limb is complete at the subsequent heel strike (D4). Adaptations in the kinematics are visible for all three joints equally (leading limb, D2). However, the ankle accomplishes the transition first (Fig 10) as the hip and the knee show substantial moment adaptations within the swing phase, though the ankle has almost no difference for level walking, transitions, and stair ambulation (always almost zero, Fig 4).

#### Trailing limb

The transition of the trailing limb (limb that enters staircase second, left, D3) begins shortly after the heel strike of D3 for all joints, with the ankle starting this transition last (Fig 10). Similar to the leading limb, large knee flexion with increased knee extension moments is found to lower the center of mass (Fig 8) with the trailing limb for the next ground contact of the leading limb. As well, the hip has reduced extension and the ankle has increased dorsiflexion to allow for lowering the center of mass. The hip flexion moment and power (Figs 4 and 7) during push off, as in level walking, are reduced. The transition of the trailing limb ends during midswing for all joints (D3).

### Stair descent to level transition

#### Leading limb

The transition from stair descent to level walking (Fig 5) begins simultaneously for the leading (limb that leaves staircase first, left, D7) and for the trailing limb (right, D8). For the leading limb, the hip and the knee begin this transition during midswing of D7, whereas the ankle transition begins in early stance of D9 (Fig 10). An increase in knee extension is found within the late swing phase (D7). Following the foot flat phase in D9, the kinetics and kinematics from stair descent transition into the level walking behavior with an increased hip range of motion, less knee flexion, and reduced ankle range of motion.

The leading limb transition is complete during the stance phase of the subsequent stride (D9). The knee performs this transition until early stance, the hip transitions until midstance, and the ankle transitions until the end of the stance (D9).

#### Trailing limb

In contrast to the leading limb (swing phase), the trailing limb begins the transition from stair descent to level walking during early midstance (D8, Fig 10). During stance, the hip begins to increase extension and the knee begins to reduce flexion (Fig 5). The ankle joint follows during early swing (D8) with a quick transfer to the level walking biomechanics.

Interestingly, there is almost no transition phase for the ankle, as the transition ends almost at the same time, whereas the hip and the knee transitions occur until the middle of the following swing (D8). Compared to the ground contact on the lower plateau of the leading limb (from D7 to D9, double stair height), no plantarflexion moments (to control the lowering of the CoM with the foot) are found for the consecutive stride of D8 (from D8 to D10, single stair height).

### Transition timing

#### Stance or swing

The overview on the transition timing reveals that the majority of transitions are initiated within the stance phase (Fig 11). For the leading limb, some transitions (hip: level to ascent; all joints: ascent to level; hip and knee: descent to level) also begin within the swing phase. For the trailing limb, only one transition begins within the swing (ankle: descent to level). The end of the transitions occurs mostly in the swing phase. However, the leading limb also completes transitions with major biomechanical changes during the stance phase.

#### Duration

The normalized (one stride) transition duration (Fig 11) is between 35% and 77% for the hip, 35% and 97% for the knee, and 1% and 92% for the ankle. In terms of means, the hip requires about 58%, the knee about 61%, and the ankle about 52%.

#### Joint level

The joint transition timings for the beginning and the end of the leading and the trailing limb are manifold (Fig 12). Among the hip, the knee, and the ankle joints, there is a maximum difference of 49% for the beginning and 35% for the end of the transition. There were also some trends identified for the timing. For both the leading and trailing limbs, the ankle initiates the level to ascent transition while the knee initiates the ascent to level transition. During stair ascent, the transition is completed last by the ankle of the leading limb. In contrast, the ankle completed the transition first for the trailing limb. During stair descent, the transition of the ankle (distal joint) always begins after the hip and/or the knee (proximal joints), whereas for the end of the transition, the transition of the ankle occurs first in all but one case (descent to level, leading limb).

## Discussion

This study analyzed four different types of gait transitions between level walking and stair ambulation. The aims of this study were threefold; (i) identify the timing of the transitions, (ii) determine if transition phases exist and determine their duration, and (iii) quantify if an order exists for the start and end of transitions for the hip, knee and ankle joints and if a time delay between joints exists.

### Transition timing

When analyzing the transition timing, we found that transitions primarily begin within stance. Thus, energy-related processes such as injection, dissipation, and redirection seem to be of major importance. For example, similar to expected walking perturbations with decreased ground level (e.g., single step), a reduction of the leg length (increased joint flexion, energy dissipation) during stance can help to prepare for a step downwards [19, 29, 30]. On the other hand, increased positive work (energy injection) during stance is required at the knee to ascend stairs [19]. We hypothesized that, if the limb that has to accomplish only one stair height, transitions could begin within the swing phase as center of mass movements could be reduced. This was only true for the leading limb hip (and almost true for the leading limb knee) for the level to ascent transition. The start within the swing was also accomplished for the trailing limb ankle (one stair height) for the descent to level transition. However, we still found a larger change in the center of mass height (Fig 8). In addition, two cases where two stair heights had to be accomplished (leading limb of the transitions for ascent to level and for descent to level) started within the swing. However, only small biomechanical changes towards the other gait mode were found within the swing phase. The major biomechanical transition happened in the following stance phase. These small but significant changes in the swing could prepare the subject for the upcoming transition or there could be methodological reasons (see section Methodological Considerations).

For most of the transitions, the transition ended in the following phase, either stance or swing, as was hypothesized. Transitions that ended in the first 4% of stance could be considered to primarily occur within the swing phase. If so, only the leading limb of the ascent to level and descent to level transitions would remain with more than one joint ending within stance. We believe that the final adaptation within the stance phase is required for the human to redirect the center of mass velocity against gravity. For the transition from level walking to ascent, the forward velocity must be redirected to match the slope of the staircase. For the transition from descent to level walking, the center of mass velocity in the direction of the staircase must be redirected to the level ground. Alternatively, for the transition from ascent to level and the transition from level to descent, gravity can assist in the redirection of the center of mass velocity, and thus these transition can end within the swing phase.

### Transition phases

The data revealed that, as hypothesized, all joints require a transition phase to realize all analyzed transitions between level walking and stair ambulation. Based on mean values, 52% to 61% of the gait cycle is required for the transition at the joint level, which demonstrates that there is no general differences between the joints. Furthermore, and as hypothesized, the transitions are completed for each limb in less than one stride, which demonstrates that humans are able to quickly adapt their gait between level walking and stair ambulation.

Based on the statistics, almost no transition phase was found for the transition from stair descent to level walking at the ankle of the trailing limb. When looking closer at the ankle angle and moment (Fig 5), this reveals a short transition phase for the angle after the toe off and a short transition phase for the moment within push off. Both were not identified to be significantly different from level walking or from stair descent.

The longest transition was required for the knee of the leading limb during the transition from stair ascent to level walking, which began in the swing phase of A7 and ended in the swing phase of A9. The late swing phase of A7 was identified to be different from stair ascent for the knee based on our statistical approach (Fig 3). Visual observation reveals that small adaptations exist but the major transition primarily occurs in stride A9.

Both extreme examples demonstrate that, when subjectively making a visual comparison of the mean values (Figs 2 to 5), extraction of the transitions using the statistical approach seems overly sensitive in one case (knee during A7) and insufficiently sensitive in another case (ankle during D8). However, the reader should consider that most figures only contain a portion of level walking (A1, D1, D11) and stair ambulation (A5 and A6 or D5 and D6) reference strides, and that there is no standard deviation of the grand means, which may also be responsible for the identified transition timings.

### Transition joint order

We found that there is large variability in the order and in the timing of the joints that begin and that end transitions, which is consistent with our hypothesis. In all but one case, all joints complete their transitions within 35% of the stride duration (Fig 12). This confirms our hypothesis of a short relative time difference. No distinct difference exists between the start and the end for the trailing and the leading limbs. This indicates that the human lower limbs, as well as the joints, have high mutual dependency, such that to perform the desired transitions, their behavior is synchronized. Such a synchronization could be the result of the human gait control or the mechanical coupling of the joints [31]. Interestingly, we could identify some trends for the joint related transition timing.

#### Transition start

The distal part of the limb (ankle and/or knee) initiates the transition for the stair ascent transitions of the leading and trailing limbs. In contrast, the proximal part of the limb (hip and/or knee) initiates the stair descent transitions. As stair ascent requires additional energy to push the CoM upwards, an assumption could be that the ankle is well suited for energy injection and for defining the support direction. Additionally, major energy injection can only happen at the ankle during stance. Comparatively, the hip and the knee are able to also inject major amounts of energy during swing to modulate the lower limb kinematics. Thus, a later start of the transition could be possible for the proximal joints. For stair descent, the foot placement based on the leg angle might be more critical than energy injection (e.g., to keep balance, for deceleration). Therefore, the hip and the knee could be the first joints that initiate the stair descent transition.

#### Transition end

During stair ascent, the transition is completed first proximally (hip) at the leading limb and distally (ankle) at the trailing limb. During stair descent, in contrast, both limbs complete the transition first distally (ankle) in all but one case. The end of the transition seems to be influenced by the nature of the joint moments. While the ankle joint moment is almost identical during the swing phase for level walking, stair ambulation, and transition strides (almost zero), it is different (shape and magnitude) in the hip and knee joints. Thus, the human can still utilize the hip and knee to change the leg behavior to another mode. As for the ankle moment, almost no differences exist within the swing phase, and as our transition identification method searches for differences in the kinematic and kinetic patterns, it will more likely find no difference there. Therefore, as most transitions end within the swing, the ankle finalizes the transitions first.

Based on our findings it becomes clear that transitions should not start and end for all joints and limbs with the same timing. Wearable lower limb robotic developers could take advantage of the observed trends in the joint specific transition timings. For example, a hip exoskeleton for gait assistance could use an angle sensor at the ankle to predict the beginning of the level to ascent transition. However, most of the other transitions can not benefit from this approach and the additional ankle angle sensors would be required. Another approach could be to use sensory information from one limb to trigger the other. As with the joint order, we can not provide a general recommendation that works for all transitions. As there is a sizeable variation, we can imagine that other detection approaches could be supplemented with information from other joints, segments, or the contralateral limb if an existing sensory infrastructure is available. As simple transition trigger approaches seem to be unavailable, we believe that advanced concepts, such as machine learning, could be a solution to identify all transitions. The presented kinematic and kinetic data, as well as unpublished data from inertial measurement units and two additional stair case heights, will serve as input and reference data to develop related transition- and gait mode-detection approaches. Detection algorithms are required to determine the gait transitions close to our identified reference timing, to be able to adapt the joint related biomechanics for transition initiation.

### Methodological considerations

Our statistical approach for the identification of the transition phases used three level walking, two stair ascent, and two stair descent strides (grand means) as references for the transition analyses. While the walking strides include a small range of walking velocities (A1: 1.25 m/s, D1: 1.01 m/s, and D11: 1.24 m/s), the stair ambulation strides include only the preferred velocity from our experiment (A5: 0.53 m/s and A6: 0.51 m/s, or D5: 0.56 m/s and D6: 0.56 m/s). Thus, the analysis is limited by not being able to account for a wider range of stair ambulation and level walk velocities. Similar to findings in [18], we believe that small, yet significant differences in the transition strides could be a reason of the walking velocity instead of being a biomechanical footprint of the terrain transition itself. Future measurements could include separate reference measurements with a range of stair ambulation and level walking velocities.

Our top plateau was limited to a length of 2.6 m. We would recommend to increase this length to at least 5 m in a future experiment so subjects could accelerate to their preferred walking velocity. In our data, it is unclear if the biomechanics of D1, which are different from A1 and D11, are a result of reduced walking velocity or if this difference is due to the terrain transition and unrelated to walking velocity. We included D1 to have a representative stride for a lower walking velocity. From a subjective visual observation of the joint angles, we believe that the transition starts in D3, and the differences in D1 (to A1 and D11) are mainly the result of walking velocity. While the hip and the knee angle of D1 are almost similar to A1 and D11, the delayed and reduced ankle plantarflexion is a typical features for lower than preferred walking velocities [3]. The joint moments also show typical features of lower walking velocities (e.g., reduced amplitudes, little shift towards the right due to prolonged stance duration). However, the shape of the ankle moment is slightly different from other level walking data of [3]. We can imagine that this is due to different methodology related to inverse dynamics, the use of a treadmill versus level ground, or also braking before the staircase compared to a cyclic walking. Stride A11 (Fig 9) is a good example where the reference data can not completely cover larger changes in walking velocity (Table 1).

The study included 12 male and no female subjects. We did not exclude female subjects on purpose but within the registration phase no female subject did register to participate. We expect no major change for our results when including female subjects, also while single biomechanical characteristics might show small but significant differences in between genders [32, 33].

Finally, some of the ends of the strides were determined by kinematic thresholds instead of vertical GRF. These thresholds were based on previous literature or determined with the help of the vertical GRF of the second stair setup, which did not contain the stance phase of this stride. As a result, it is possible that strides were not segmented with the same timing as when segmented based on the vertical GRF (too late and/or early). Timing differences will result in shifts of the extracted stride-based data and an increased standard deviation in between subjects, which would result in less accurate transition timings. Thus, in an ideal case, future measurements with a comparable purpose should use more force plates to cover all strides (five on top, five on bottom, five on the stairs).

## Conclusions

This study analyzed the lower limb joint biomechanics of four types of gait transitions between level walking and stair ambulation to identify the timing of the transitions, to determine if transition phases exist and how long they last, and to investigate if there exists a joint-related order and timing for the beginning and ending of the transitions.

We found that, regarding the timing, transitions primarily begin within the stance phase and end within the swing phase. While the stance phase primarily allows for changes in the energy, the swing phase can be used for kinematic adaptations. Two transitions were also found to terminate in the stance phase. We believe that the ending within the stance is required to redirect the center of mass, against the gravity, in the walking direction.

Furthermore, we found that transition phases exist for all joints and types of transitions, and that they last, on average, approximately half of one stride for each limb. No limb-specific transition was longer than one stride.

Regarding the joint-related order, we found that differences between the beginning and end timing of all joints of a transitioning limb are, for all but one case, shorter than 35% of the stride duration and that no general order exists for all transitions. However, some trends were identified. Primarily, distal joints initiate the stair ascent and proximal joints the stair descent transitions. We believe that the ankle is of critical importance during stair ascent to inject the required energy while the proximal hip could be of importance to control the balance. The end of the transitions are primarily completed first by the ankle. It is assumed that this occurs as most of the transitions end within the swing phase where the hip and knee kinetics can still be manipulated to redirect the lower limb.

Regarding the finding that there exists a transition phase for all joints and transitions, we believe that wearable lower limb robotic control approaches should be able to account for these transitions. This could lead to improved smoothness in transition and consequently, increased comfort, safety, and an improved user experience.

Furthermore, we believe that our results could be used to design wearable lower limb robotic control approaches that result in similar gait characteristics as those identified for steady gait and for transition phases. The concepts could be compared and evaluated based on our findings.

As the variety of the transitions (four transitions, three joints, two limbs) and their joint-related order in timing are manifold, designers should evaluate if some of the identified joint-related trends can be used to trigger a transition. We found, however, that no simple solution exists that is suitable for all transitions. We therefore believe that detection concepts could include machine learning methods, trained with transition kinematics (e.g., joint or segment angles and velocities), which can be measured online in a robotic assistance device.

## Supporting information

S1 Fig Marker placement. Location of the motion capture markers.
(TIF)

S1 Table Location names of the motion capture markers. The r and the l indicate the right and the left side, respectively.
(PDF)

S1 Data (MAT)
