# Supplementary figures and images for: Lower limb joint biomechanics-based identification of gait transitions in between level walking and stair ambulation
Source: PLoS One. 2020 Sep 16;15(9):e0239148. doi: 10.1371/journal.pone.0239148 (PMC7494088; doi:10.1371/journal.pone.0239148)

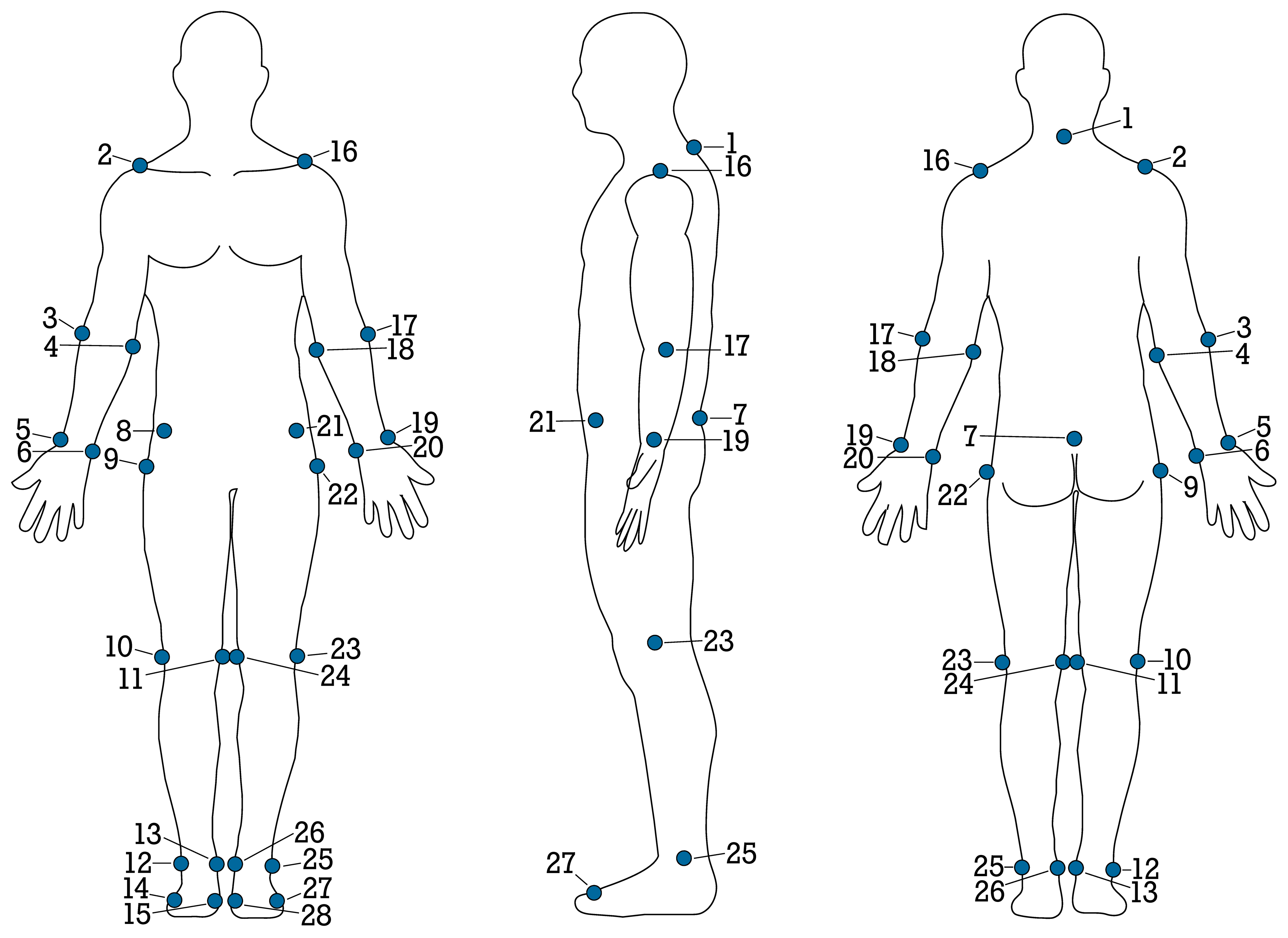

Supplement: S1 Fig — Location of the motion capture markers. (TIF) [file pone.0239148.s001.tif]
