# Supplementary material for: Lower limb joint biomechanics-based identification of gait transitions in between level walking and stair ambulation
Source: PLoS One. 2020 Sep 16;15(9):e0239148. doi: 10.1371/journal.pone.0239148 (PMC7494088; doi:10.1371/journal.pone.0239148)
Supplement: S1 Table — The r and the l indicate the right and the left side, respectively. (PDF) [file pone.0239148.s002.pdf]

S1 Table. Location names of the motion capture markers. The r and the l indicate the right and the left side, respectively.

Motion Capture Marker	
#	Location
1	Vertebra prominens (C7)
2	r. Acromion
3	r. Epicondylus lateralis humeri
4	r. Epicondylus medialis humeri
5	r. Processus styloideus radii
6	r. Processus styloideus ulnae
7	Sacrum
8	r. Spina Iliaca anterior superior
9	r. Trochanter Major
10	r. Condylus lateralis femoris
11	r. Condylus medialis femoris
12	r. Malleolus lateralis
13	r. Malleolus medialis
14	r. Articulationes metatarsophalangeae V (Mt5)
15	r. Articulationes metatarsophalangeae I (Mt1)
16	l. Acromion
17	l. Epicondylus lateralis humeri
18	l. Epicondylus medialis humeri
19	l. Processus styloideus radii
20	l. Processus styloideus ulnae
21	l. Spina Iliaca anterior superior
22	l. Trochanter Major
23	l. Condylus lateralis femoris
24	l. Condylus medialis femoris
25	l. Malleolus lateralis
26	l. Malleolus medialis
27	l. Articulationes metatarsophalangeae V (Mt5)
28	l. Articulationes metatarsophalangeae I (Mt1)
